# Delayed Bone Maturation and Extended Growth Phase as Distinctive Features of 17α‐Hydroxylase/17,20‐Lyase Deficiency: A Retro‐Prospective Study of a Large Patient Cohort

**DOI:** 10.1111/cen.15261

**Published:** 2025-05-11

**Authors:** Rafaela Fontenele, Flávia A. Costa‐Barbosa, Marivânia Costa‐Santos, Rafael L. Batista, Lívia M. Mermejo, Berenice B. Mendonça, Margaret de Castro, Gil Guerra‐Júnior, Claudio E. Kater, Brazilian Congenital Adrenal Hyperplasia Multicenter Study Group (BCAHMSG)

**Affiliations:** ^1^ Division of Endocrinology and Metabolism, Department of Medicine School of Medicine and BCAHMSG Federal University of São Paulo São Paulo Brazil; ^2^ Discipline of Endocrinology & Metabolism, Department of Internal Medicine University of Sao Paulo Medical School University of Sao Paulo Sao Paulo Brazil; ^3^ Department of Medicine Ribeirao Preto Medical School University of Sao Paulo Sao Paulo Brazil; ^4^ Growth and Development Laboratory Center for Investigation in Pediatrics (CIPED), School of Medical Sciences University of Campinas (UNICAMP) Campinas Brazil

**Keywords:** bone retardation, congenital adrenal hyperplasia, CYP17A1, growth and development, hypogonadism, P450c17, tall stature

## Abstract

**Introduction:**

Worldwide, combined 17‐hydroxylase/17,20‐lyase deficiency (CYP17D) is a rare form of congenital adrenal hyperplasia, but it is the second most prevalent type in Brazil. An absence of sexual differentiation and hypergonadotropic hypogonadism arise from a reduction in the usual pattern of sex steroid formation in the adrenals and the gonads, and virtually all affected individuals are phenotypically female, regardless of karyotype. The absence of sex steroids precludes bone maturation, allowing an extended growth phase, such that nontreated adult patients usually have a tall eunuchoid appearance. Mineralocorticoid hypertension is an associated feature.

**Objective:**

To describe the clinical aspects of growth development, bone maturation, and body proportions of a large cohort of Brazilian patients with CYP17D.

**Patients and Methods:**

The study involved an analysis of the records of 88 patients with CYP17D who were treated at the Federal University of São Paulo Medical School and other Endocrine Reference Centres in Brazil.

**Results:**

At diagnosis, the median chronological age and bone age of non‐adult patients were 15.8 years (range: 10–20 years; *n* = 41) and 11 years (7.5–15 years; *n* = 25), respectively. A delay of ≥ 2 years in bone age was present in 92.5% of cases. In 30 patients, the height and its *Z*‐score were 157 cm (130–171.5 cm) and −0.4 (−3.0 to +1.6), respectively. The span‐to‐height ratio was high and consistent over time. Final heights were available for 51 patients, of which 77% (25 XY, 14 XX) were in the 50th percentile or higher, and 39% (14 XY, 6 XX) were in the 90th percentile or higher. Only 8% (1 XY, 3 XX) were in the 25th percentile or lower. Of the 42 patients with data available, 11 (26%) had lower *Z‐*scores during childhood and adolescence, and it is plausible that they missed a growth spurt.

**Conclusion:**

In this large CYP17D cohort, we verified that the prolonged hypoestrogenism that led to delayed or absent puberty was associated with decreased bone age, lower stature in childhood and adolescence, missed growth spurts, an extended growth phase, and greater final heights with frequent eunuchoid appearance.

## 1 Introduction

17α‐hydroxylase deficiency (CYP17D) was first recognised by Biglieri et al. in 1966 [1]. The index case was a 35‐year‐old phenotypic XX female patient with hypogonadism and severe hypertension who was nearly six feet tall. Worldwide, CYP17D is considered a rare form of congenital adrenal hyperplasia (CAH), accounting for less than 1% of cases. Nevertheless, it is the second most prevalent form of CAH in Brazil, representing nearly 7% of cases [2, 3]. The *CYP17A1* gene encodes for a single 17α‐hydroxylase enzyme (CYP17A1). The enzyme is expressed in both the adrenal cortices and the gonads [4] and catalyzes two sequential reactions: the 17α‐hydroxylation of both progesterone and pregnenolone and the 17,20‐side‐chain cleavage (17,20‐lyase) of its products.

CYP17A1 is responsible for cortisol synthesis in the adrenal cortex and sex‐steroid formation in the adrenals and the gonads. Total loss of enzyme activity leads to the complete and most prevalent form of the disease [1, 3, 5, 6, 7]. Impaired production of oestrogens or androgens results in an absence of sexual differentiation and a state of hypergonadotropic hypogonadism (HH) with elevated progesterone levels and associated mineralocorticoid hypertension due to excess deoxycorticosterone (DOC). These effects are typically evident around the expected time of puberty. Isolated 17,20‐lyase deficiency, which is associated with only HH, is an even rarer condition [8].

Genotypic XX and XY patients are virtually all phenotypically female due to impaired production of sex steroids and absent differentiation of the external genitalia. Consequently, they all have pubertal failure with an absence of secondary sex characteristics (sexual infantilism) and primary amenorrhoea [3, 9, 10, 11]. As a result of lacking sex‐hormones, particularly because of the long‐standing hypoestrogenic state, untreated patients with CYP17D present delayed bone maturation, extended linear growth before epiphyseal fusion, an absence of growth spurts, tall stature, and frequent eunuchoid appearance in adulthood [3, 9, 10, 11].

There have been few reports of detailed clinical evaluations of the growth aspects and bone development patterns in patients with CYP17D. Thus, the aim of the present study is to examine the impact of the reduction in the usual pattern of sex‐steroid and cortisol formation upon growth development and bone maturation from initial diagnosis in infancy and adolescence throughout adulthood. We examined a large cohort of Brazilian patients with a confirmed diagnosis of CYP17D and correlated final heights with familial target heights, as well as the influence of oestrogen replacement.

## 2 Patients and Methods

We analysed data from the medical records of 88 patients who were clinically, hormonally, or genotypically confirmed as having CYP17D, including 41 XY cases, 35 XX cases, and 12 cases in which the karyotype was not reported. This retro‐prospective study examined an 11‐year period and included patients treated at the Division of Endocrinology and Metabolism of the Federal University of São Paulo Medical School, as well as another 21 Endocrine Reference Care Centres for CAH in Brazil. All centres agreed to collaborate after receiving a formal invitation.

Specific physicians from each center were responsible for the collection, verification, and forwarding of data to our center on individual spreadsheets for data analysis. However, complete sets of data were not available for every patient. Information collected at the most recent visit to the respective centres included chronological age (CA) and bone age (BA) in years, as well as height and span (cm). We calculated the standard deviations of height (*Z‐*scores), span‐to‐height ratios, height *Z‐*score‐to‐CA ratio, and CA‐to‐BA ratio. BA was estimated using the Greulich and Pyle method [12] from X‐ray images of the nondominant wrist, and *Z‐*scores for height were calculated using Tanner charts [13].

A span‐to‐height ratio ≥ 1 was considered as high and indicative of an eunuchoid body proportion or appearance [14], and BA was considered delayed if it was ≥ 2 years behind CA [15]. To assist with estimations patients with CA > 20 years and complete bone epiphyseal fusion as adults in accordance with the theoretical basis in the literature on bone maturation disorders [16] and in consideration of the profile of the study population. We compared final adult‐height *Z‐*scores with mid‐parental target‐height *Z‐*scores for females for the 42 individuals with available data. The target height (in cm) was calculated as follows: (mother's height + father's height – 13)/2.

Bone mineral density (BMD) was obtained from 35 patients at the time of diagnosis. Blood levels of luteinizing hormone (LH), follicle‐stimulating hormone (FSH), estradiol, progesterone, cortisol, and adrenocorticotropic hormone (ACTH) at diagnosis were analysed when available. The levels were determined by different laboratories, which mostly used chemiluminescence methods.

For eight of the 88 enroled patients (9.1%), height, BA, CA, and derived data were obtained before the final diagnosis of CYP17D. CA and BA were available for 57 and 38 of the 88 patients at diagnosis, respectively. Since not all data were obtained for all patients, parameters are presented for different numbers of patients.

Statistical analyses were performed using the software package SPSS 16.0. Descriptive statistics were used to evaluate clinical characteristics, and variables were represented as the mean ± standard deviation (SD) or the median and range, depending on the normality of their distributions. Student's *t‐*test was used to compare the means of two independent groups. The chi‐squared test was used for comparing categorical variables, and Pearson's correlation was used to analyse BA and CA. A value of *p* < 0.05 was considered statistically significant.

The study was conducted in accordance with the Declaration of Helsinki (1975) and was approved by the Committee of Ethics in Research of the Federal University of São Paulo (protocol #146.087, 14 November 2012).

## 3 Results

### 3.1 Data at Diagnosis

Data at diagnosis were obtained from 57 of the 88 patients, which included 41 non‐adults (≤ 20 years) and 16 adults ( > 20 years). As shown in Table 1, the median CA of non‐adult patients was 15.8 years (10–20 years), and that of adults was 27 years (22–40 years). The median CA of the whole cohort was 16.5 years. The median height and corresponding *Z‐*score of 30 non‐adults were 157 cm (range 130–171.5 cm) and −0.4 (−3.0 to +1.6), respectively, whereas those of 14 adults were 169.8 cm (158–191 cm) and +1.6 (−0.3 to +4.9).

**TABLE 1:** Clinical data of patients with 17α‐hydroxylase deficiency at diagnosis (split in non‐adults [≤ 20 y] and adults [> 20 y]) and at the most recent visit (current; all adults).

	At diagnosis				Current	
	Non‐adults (≤ 20 y)	N	Adults (> 20 y)	N	All adults (> 20 y)	N
Age (CA, y)	15.8 (10–20)	41	27 (22–40)	16	40.5 (20–66)	56
Height (cm)	157 (130–171.5)	30	169.8 (158–191)	14	170 (154–191)	51
Height Z‐Score	−0.4 (−3.0 to +1.6)	30	+1.6 (−0.3 to +4.9)	14	+1.3 (−1.3 to +4.8)	51
Span/Height ratio	1.03 (0.93–1.18)	19	1.0 (0.98–1.08)	8	1.02 (0.98–1.08)	25
Bone Age (BA, y)	11 (7.5–15)	27	Adult	11	Adult	37
CA/BA ratio	1.42 (1.2–2.07)	27	Adult	11	Adult	37

*Note:* Data are presented as median and range.

**FIGURE 1:**
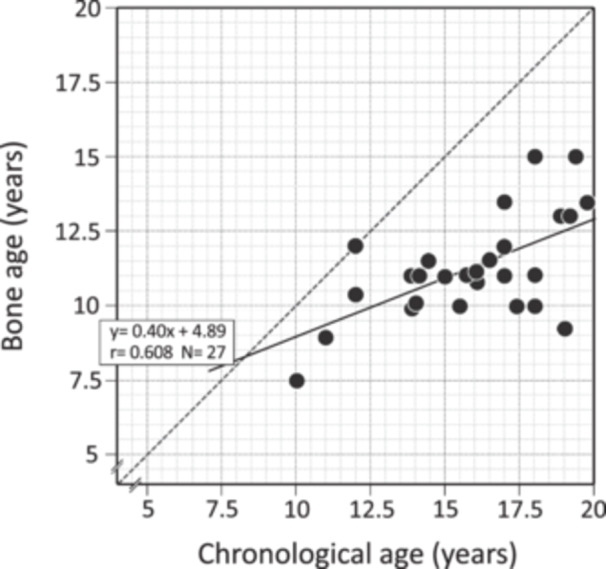
Individual bone ages plotted against chronological ages in patients with CYP17D at the time of diagnosis.

There were 38 patients (11 adults and 27 non‐adults) who had concurrent data on CA and BA at diagnosis. All adults had closed epiphyseal bones (adult BA), and 25 of the non‐adults (92.6%) showed delayed BA ( > 2 years) (median BA delay = 5.0 years; range 2.2–9.8 years) (Figure 1). There was a significant positive correlation between BA and CA (*y* = 0.40*x* + 4.89, *r* = 0.608*. n* = 27, *p* < 0.01), but it was considerably below the identity line (Figure 1).

BMD was available for 35 patients (all adults) at the time of diagnosis and indicated a significant impairment of bone mass. The adjusted median *Z‐*scores for BMD were −2.10 (range: −4.13 to +0.25). Detailed results regarding bone mineral characteristics will be reported elsewhere.

**FIGURE 2:**
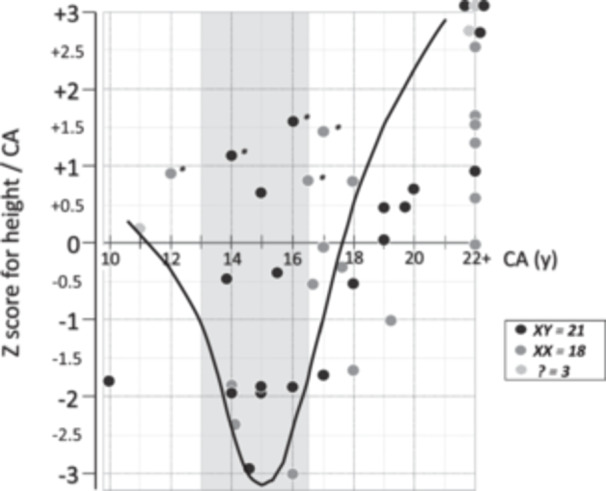
Individual Z‐scores for height/CA in patients with CYP17D at the time of diagnosis. Observe departure from baseline across adolescence (possibly missed growth spurt) (curved line) and catch‐up during adulthood. Patients designated with an asterisk have already been treated with oestrogens.

The ratios of arm span to height were high and virtually unchanged over time (Table 1). Several patients diagnosed during childhood and adolescence were in the lower range of *Z‐*score for the height/CA ratio, indicating an obvious missed growth spurt (Figure 2). Conversely, when diagnosis was established in late adolescence and early adulthood, the *Z‐*score for the height/CA ratio was normal or high, suggesting late catch‐up growth. Additionally, no difference occurred in the *Z‐*score for the height/CA ratio when patients were evaluated by karyotype (Figure 2).

### 3.2 Most Recent Data

**FIGURE 3:**
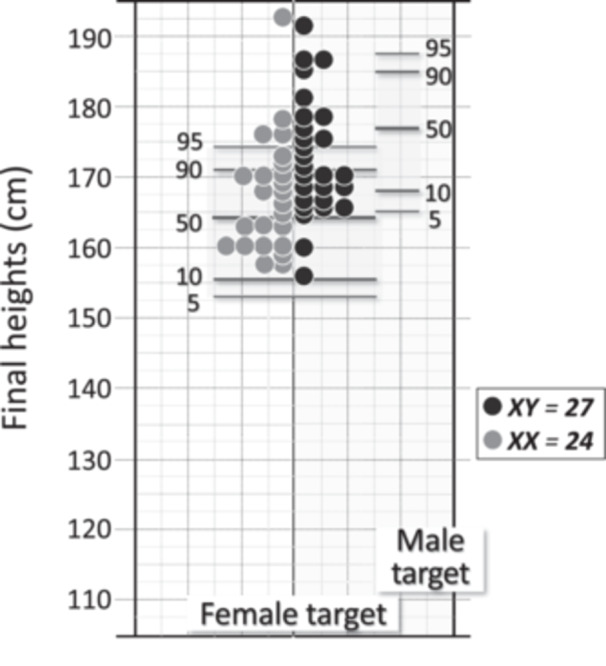
Individual final heights of 51 adult patients ( > 20 years of age and closed epiphyseal bones) with CYP17D (27 XY/24 XX) plotted against a standard target height scale for female phenotype (target height for adult males are also shown on the right, for comparison).

In 56 of the 88 patients, the median age and height at the most recent visit were 40.5 years (19–66 years) and 170 cm (154–191 cm), respectively, and the final height *Z‐*score was +1.3 (−1.3 to +4.8) (Table 1). Individual final heights were plotted against a standard target height scale for females, and 76.5% of the patients (39/51) were in the 50th percentile or higher, 39% (20/51) were in the 90th percentile or higher, and only 7.8% (4/51) were in the 25th percentile or lower. When height was evaluated according to karyotype (27 XY, 24 XX), 48% of XY cases were above the 90th percentile, whereas 25% of XX cases were above this percentile (Figure 3). However, when XY patients were plotted against a standard target height scale for males, only 30% of them (8/27) were in the 50th percentile or higher.

**FIGURE 4:**
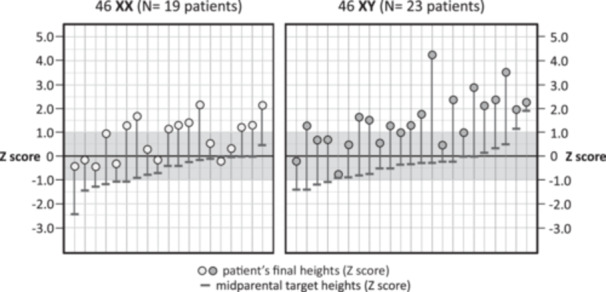
Final individual adult height Z‐scores of 42 patients with CYP17D (19 XX/23 XY) (dots) plotted together with the respective mid‐parental target height Z‐scores (dashes), calculated based on female standard ([mother height + father height, −13] divided by 2, in cm).

Most patients received their final diagnosis in adulthood, but among those diagnosed at 18 years of age or younger (*n* = 25), the majority (18/25) had lower *Z‐*scores for height. Only 7/25 were above +0.5 during childhood and throughout adolescence (Figure 2). Figure 4 shows the final *Z‐*scores for height of each patient in comparison to the mid‐parental target heights (calculated for females). All but one of the 42 patients achieved final statures above the mid‐parental targets for females.

### 3.3 Hormonal Aspects

Blood levels of gonadotropins, sex steroids (estradiol, progesterone), cortisol, and ACTH were available at diagnosis from several nontreated patients (Table 2). The gonadotropin values were typical of a secondary hypergonadotropic (or 'menopausal') state: 74.9 IU/L (20–164) for FSH and 37 IU/L (21.8–40) for LH. The serum progesterone concentrations were also significantly increased at 600 ng/dL (100–1280), whereas estradiol was consistently reduced or undetectable. Cortisol was reduced, and ACTH was increased in virtually all patients evaluated (*n* = 30 and *n* = 16, respectively; Table 2).

**TABLE 2:** Hormonal data (as mean ± SD, and median and range) at diagnosis from untreated patients with 17α‐hydroxylase deficiency.

	Mean ± SD	Median (range)	Reference range^a^	N
FSH (IU/L)	77.5 ± 33.6	74.9 (20–164)	3.5–12	28
LH (IU/L)	54.9 ± 46	37 (21.8–40)	2.4–12	28
Estradiol (pg/mL)	< 12	< 12 (< 12–21)	12–166	20
Progesterone (ng/dL)	614 ± 290	600 (100–1280)	20–150	25
ACTH (pg/mL)	186.4 ± 133.2	122 (18–476)	7–63	16
Cortisol (µg/dL)	2.1 ± 2.9	0.9 (0.1–10.1)	7–25	30

^a^ Reference ranges for the follicular phase, when applicable. To convert metric to SI units: (a) pg/mL to nmol/L: multiply estradiol by 3.67 and ACTH by 0.22; (b) ng/dL to nmol/L: multiply progesterone by 0.0318; (c) μg/dL to nmol/L: multiply cortisol by 27.6.

## 4 Discussion

The present study examined long‐term clinical data on the linear growth and body proportions of a large cohort of Brazilian patients with CYP17D. We were able to obtain data from a total of 88 patients from different centres and private clinics from all regions of Brazil, although complete sets of data were not available for every patient. The median age at diagnosis of the whole cohort was 16.5 years, which is much younger than the age of 35 years of the index case reported by Biglieri et al. [1] This hereditary disease is still highly underdiagnosed [9], even though virtually all reported cases exhibit the quite distinctive clinical manifestations of the disease, such as hypergonadotropic hypogonadism and mineralocorticoid‐type hypertension.

Interestingly, patients who were diagnosed at a prepubertal age (26.2%) were in the normal/low range of the *Z‐*score for the height/CA ratio. This contrasts with those who were diagnosed later toward adolescence or adulthood, when significant catch‐up growth has already occurred (as shown in Figure 2). Accordingly, most of these patients have attained a tall stature and the highest percentiles of final heights (39% were in the 90th percentile or higher). Although all patients were phenotypically and socially females, it is noteworthy that the highest statures were observed in the XY patients. This observation suggests that growth development or final stature of these patients may be subject to additional factors other than sex hormones, which may possibly be linked to the Y chromosome. This pattern has also been observed in patients with Klinefelter's syndrome [17, 18].

Linear bone growth (LBG) during the prepubertal period primarily depends on normal secretion of growth hormone (GH) and somatomedins. However, physiological production of oestrogen during the prepubertal stage may enhance skeletal growth and positively affect LBG [19, 20, 21, 22]. In Turner syndrome, for instance, growth hormone and low‐dose oestrogen enhance LBG [23], while ultra‐low doses of oestrogens administered to prepubertal patients demonstrate a clear growth‐promoting effect [24]. Thus, we also expect oestrogen deficiency to contribute to the lower *Z‐*score for height/CA ratios in the prepubertal period as well.

Most of our patients did not develop a pubertal growth spurt. Consequently, they might have been predisposed to tall statures and eunuchoid body proportions due to their extended linear growth into adulthood, which is associated with delayed bone maturation [25]. This has also been observed in other conditions with hypoestrogenism, such as aromatase deficiency, oestrogen resistance, and idiopathic hypogonadotropic hypogonadism [26, 27, 28, 29]. Furthermore, due to oestrogen deficiency, virtually all patients in this series had a low bone mass, as demonstrated by their reduced BMD *Z‐*scores. There are scarce data on series of patients with CYP17D for comparison of these aspects of growth, but tall stature and eunuchoid appearance are consistently mentioned in several isolated reports [1, 3, 5, 30, 31, 32, 33, 34, 35, 36, 37], although there is also an unexpected report of a patient with short stature [38].

Notably, both tall stature and the eunuchoid phenotype may be offset by early sex‐hormone replacement therapy [19, 22]. Findings from some of our patients are consistent with this and show that dysmorphic final body proportions did not occur for patients who were diagnosed at earlier ages (usually during family screening) or because of a preliminary diagnosis of hypogonadism and had timely sex‐hormone replacement. Accordingly, recent data reported by Siklar et al. [39] have shown that final adult heights and normal span‐to‐height ratios were normal among a large cohort of Turkish patients with CYP17D. In contrast to our study, however, their patients were diagnosed in paediatric endocrine centres at an early age (53% at < 10 years and 10% at < 5 years of age). Furthermore, all of their patients received prompt glucocorticoid (GC) therapy at diagnosis and proper oestrogen replacement at the time of puberty. These findings provide evidence that classic characteristics related to body proportions in CYP17D may not manifest if patients are diagnosed and properly treated before puberty.

GC receptors have been identified in hypertrophic chondrocytes in the human growth plate, and it is well documented that they exert profound effects on many skeletal growth factors and cytokines, which suggest that they have direct effects of GCs on growth plate function [40, 41, 42, 43]. Growth disturbance may occur among children with primary adrenal insufficiency, which is minimised by GC and mineralocorticoid replacement [42, 43]. Despite patients with CYP17D showing a 20‐fold increase in the levels of corticosterone (which has 20% of cortisol's activity), the impaired production of cortisol may still play a role in the initial short stature (lower range of *Z‐*score for height/CA ratios) observed in several prepubertal patients at diagnosis. If not excessive, GC replacement usually leads to normal adult body proportions.

The XY patients in this cohort were taller than the XX patients in adulthood. This observation is consistent with findings that individuals with XY gonadal dysgenesis tend to be taller than those with XX chromosomes [44]. Furthermore, boys with 47 XYY chromosomes are taller compared to those with 47 XXY [17, 18, 44]. The final heights of XY females with androgen insensitivity syndrome (AIS) are generally greater than average for females, and their adult heights fall between those of typical males and females [45, 46]. Similarly to AIS, the lack of androgens in CYP17D could lead to the reduced final adult height of XY patients [45]. Therefore, it is conceivable that the presence of additional growth‐related genes or other factors in the Y chromosome may be responsible for these gender differences in height, but no specific candidates have been identified thus far [17, 44].

Although the number of patients in this study was considerable, we were not able to gather complete sets of pertinent data from all of them since patients were treated at several clinical centres throughout Brazil, which each have their own routine and difficulties. Thus, retrospective data from charts were not necessarily available. In particular, data were rather scarce in regard to patient's karyotypes and the possible use, dosage, and timing of oestrogen or progestin treatment before the final diagnosis. This information could have helped to explain the subgroup of patients who did not have increased final heights or ratios of arm span to height.

There are a number of interplaying factors that were not evaluated in the present study but may have influenced the individual final heights of patients with CYP17D. Examples include nutritional status, inflammatory cytokines, paracrine growth factors, extracellular matrix factors, and intracellular proteins [47, 48]. Likewise, it is important to consider that in CYP17D, impairments in full growth capability are also affected by concurrent clinical features such as limited cortisol production (which is partially compensated for by increased corticosterone levels), long‐standing moderate to severe hypertension, and persistent increased susceptibility to infection.

In summary, our findings elucidate some phenotypic characteristics of individuals with CYP17D concerning stature, body proportions, growth rate, and bone maturation. At diagnosis, long‐standing hypoestrogenism, as well as belated or poorly controlled oestrogen treatment and GC replacement, lead to untreated CYP17D patients showing similar features of female social sex, delayed or absent puberty, and delayed BA. Most were short before puberty and possibly missed growth spurts but reached normal to tall stature afterwards. Untreated adult patients systematically had greater final heights than the family target, as well as eunuchoid body proportions. The appropriate use of oestrogen replacement by a few patients some time before the final diagnosis could have precluded both a tall stature and the eunuchoid appearance.

## Brazilian Congenital Adrenal Hyperplasia Multicenter Study Group (BCAHMSG)

Adriane M. Rodrigues (Endocrine and Metabolism Center at the Federal University of Paraná ‐ SEMPR/UFPR); Alberto Aloysio Larcher de Almeida (Santa Casa de Misericórdia de Juiz de Fora, MG); Ayrton C. Moreira (University of São Paulo Medical School at Ribeirão Preto ‐ FMUSP‐RP)**;** Bernardo Liberman (University of São Paulo Medical School at São Paulo ‐ FMUSP‐SP); Carolina Leães Rech (Endocrinology and Paediatric Endocrinology, Department of Medicine at UFCSPA, RS); Cristiana Pontes (National University of Brasilia Medical School at Brasilia); D.L. Queiroz (Private Clinic); Eduardo P. Dias (Felício Rocho Hospital, Federal University of Minas Gerais, UFMG); Eliana Ternes Pereira (Private Clinic); Fabiano Sandrini (Private Clinic, Cascavél, PR); Gisele Toyama (Private Clinic, Cascavél, PR); Ikaro Soares S. Breder (State University of Campinas, São Paulo ‐ UNICAMP); Ivan Ascêncio (Private Clinic, Mogi das Cruzes, SP); Ivan R.A. Ferreira (Private Clinic, São Paulo, SP); João Paulo B. Vieira‐Filho (Federal University of São Paulo ‐ UNIFESP, SP); José Gastão R. Carvalho (Endocrine and Metabolism Center at the Federal University of Paraná ‐ SEMPR/UFPR); Luiz de Lacerda Filho (Endocrine and Metabolism Center at the Federal University of Paraná ‐ SEMPR/UFPR); Manuel H. Aguiar‐Oliveira (Endoclínica Pio XI, Aracaju, SE); Marcia Helena Costa (UNIRIO, Rio de Janeiro, RJ); Maria Conceição R. Freitas (Getúlio Vargas Hospital ‐ HGV, Recife, PE); Maria Tereza M. Baptista (Private Clinic, Cascavél, PR); Mário Sérgio Almeida (National University of Brasilia Medical School at Brasilia); Marisa Cesar Coral (Federal University of Santa Catarina ‐ UFSC); Marta Sarquis Soares (Felício Rocho Hospital, Federal University of Minas Gerais, UFMG); Mauro A. Czepielewski (Hospital de Clínica de Porto Alegre, Federal University of Rio Grande do Sul ‐ HCPA/UFRS); Mauro Dinato (Private Clinic, Santos, SP); Mauro Semer (Hospital Brigadeiro, São Paulo, SP); Milena F. Caldato (CESUPA ‐ Belém, Pará); Monike L. Dias Rodrigues (Federal University of Goiás ‐ UFGO); Ney Cavalcanti (Getúlio Vargas Hospital ‐ HGV, Recife, PE); Rosângela R. Rea (Endocrine and Metabolism Center at the Federal University of Paraná ‐ SEMPR/UFPR); Suely dos Santos (Federal University of Goiás ‐ UFGO); Suzana Pacheco (Hospital do Servidor Público Estadual ‐ HSPE, São Paulo, SP); Theresa Selma S. Lins (Private Clinic, Recife, PE); and Thomaz R.P. Cruz (Federal University of Bahia ‐ UFBA, Salvador, BA).
